# Aspartame, as an artificial sweetener, does not affect renal function and antioxidative states in mice

**DOI:** 10.1186/s13104-024-06816-6

**Published:** 2024-06-05

**Authors:** Kenta Torigoe, Miki Torigoe, Satoru Oka, Yoko Obata, Hiroshi Mukae, Tomoya Nishino

**Affiliations:** 1 Department of Nephrology, Nagasaki University Hospital, 1-7-1 Sakamoto, Nagasaki, 852- 8501 Japan; 2 Department of Respiratory Medicine, Nagasaki University Graduate School of Biomedical Sciences, 1-7-1 Sakamoto, Nagasaki, 852-8501 Japan

**Keywords:** Aspartame, Kidney, Food safety

## Abstract

**Background and objective:**

Aspartame (l-aspartyl l-phenylalanine methyl ester) is an artificial sweetener widely used as a sugar substitute. There are concerns regarding the effects of high aspartame doses on the kidney owing to oxidative stress; however, whether the maximum allowed dose of aspartame in humans affects the kidneys remains unknown. Therefore, in this study, we investigated whether the maximum allowed dose of aspartame in humans affects the kidneys.

**Methods:**

In this study, animals were fed a folate-deficient diet to mimic human aspartame metabolism. Eight-week-old ICR mice were divided into control (CTL), 40 mg/kg/day of aspartame-administered (ASP), folate-deficient diet (FD), and 40 mg/kg/day of aspartame-administered with a folate-deficient diet (FD + ASP) groups. Aspartame was administered orally for eight weeks. Thereafter, we evaluated aspartame’s effect on kidneys via histological analysis.

**Results:**

There were no differences in serum creatinine and blood urea nitrogen levels between the CTL and ASP groups or between the FD and FD + ASP groups. There was no histological change in the kidneys in any group. The expression of superoxide dismutase and 4-hydroxy-2-nonenal in the kidney did not differ between the CTL and ASP groups or the FD and FD + ASP groups.

**Conclusion:**

Our findings indicate that the allowed doses of aspartame in humans may not affect kidney function or oxidative states.

**Supplementary Information:**

The online version contains supplementary material available at 10.1186/s13104-024-06816-6.

## Introduction

Artificial sweeteners are food additives that provide a sweet taste without adding additional calories. Aspartame (l-aspartyl l-phenylalanine methyl ester) is a type of artificial sweetener discovered by James Schlatter in 1965 [1]. It is one of the most used artificial sweeteners worldwide, with an annual consumption of 16,000 tons [2]. Based on the safety evaluation of aspartame, the Food and Drug Administration set its maximum daily intake (ADI) at 50 mg/kg/day. The European Union set it at 40 mg/kg/day [3, 4]. Aspartame is present in various foods consumed daily, and its consumption is likely to continue rising. However, there are concerns regarding the safety of aspartame in obesity, diabetes mellitus, children and fetuses, autism, neurodegeneration, phenylketonuria, allergies, and skin problems, as well as its carcinogenic properties and genotoxicity [1].

The side effects of aspartame on the kidneys have been investigated in animal experiments and reported to cause damage to the glomeruli and renal tubules, as well as renal dysfunction [5]. The mechanism through which aspartame affects the kidneys is thought to involve its metabolites. Once ingested, aspartame is metabolized to aspartic acid, phenylalanine, and methanol in a 50:40:10 ratio [6]. Methanol is further broken down into formaldehyde and formic acid [7], which cause a decrease in antioxidant substances and an increase in oxidative stress, potentially leading to kidney damage [5, 8]. Rodents metabolize methanol quickly because of the abundance of folic acid in their livers and may have different tolerances to aspartame than humans [9]. Therefore, in most animal experiments, the effects of aspartame on kidneys have been investigated at doses higher than the maximum doses allowed in humans. However, to examine aspartame nephrotoxicity in humans more thoroughly, conducting studies under conditions that closely mimic human aspartame metabolism is necessary.

Therefore, in this study, we used mice fed a folate-deficient diet to examine whether the allowed doses of aspartame in humans affect the kidneys.

## Materials and methods

### Animals

Animal experiments were conducted using eight-week-old male ICR mice (Japan SLC Inc., Shizuoka, Japan). The mice were housed in standard rodent cages in a light- and temperature-controlled room at the Biomedical Research Center, Center for Frontier Life Sciences, Nagasaki University (Nagasaki, Japan), and had free access to laboratory food and tap water. The room was maintained at a temperature of 21–25 °C (without sudden changes) and a humidity of 40–70% according to the facility’s regulations. The lighting in the animal housing room was set to turn on at 7:00 AM and turn off at 7:00 PM. The experimental protocol was evaluated by the Animal Care and Use Committee of Nagasaki University and approved by the President of Nagasaki University (Approval number: 2005011627-7).

### Animal experimental protocol

In this study, the mice were fed a folate-deficient diet to mimic human methanol metabolism. A folate-deficient l-amino acid rodent diet supplemented with 1% succinyl sulfathiazole (Cat. no. 517777; Dyets, Inc., Bethlehem, PA, USA) was used. Previous reports have confirmed that plasma and liver folic acid levels decrease when ICR mice are fed this diet [10]. In our pilot study, we confirmed that plasma folic acid levels were depleted in ICR mice after maintenance on a folate-deficient diet for four weeks (Supplemental Fig. 1).

**Fig. 1:**
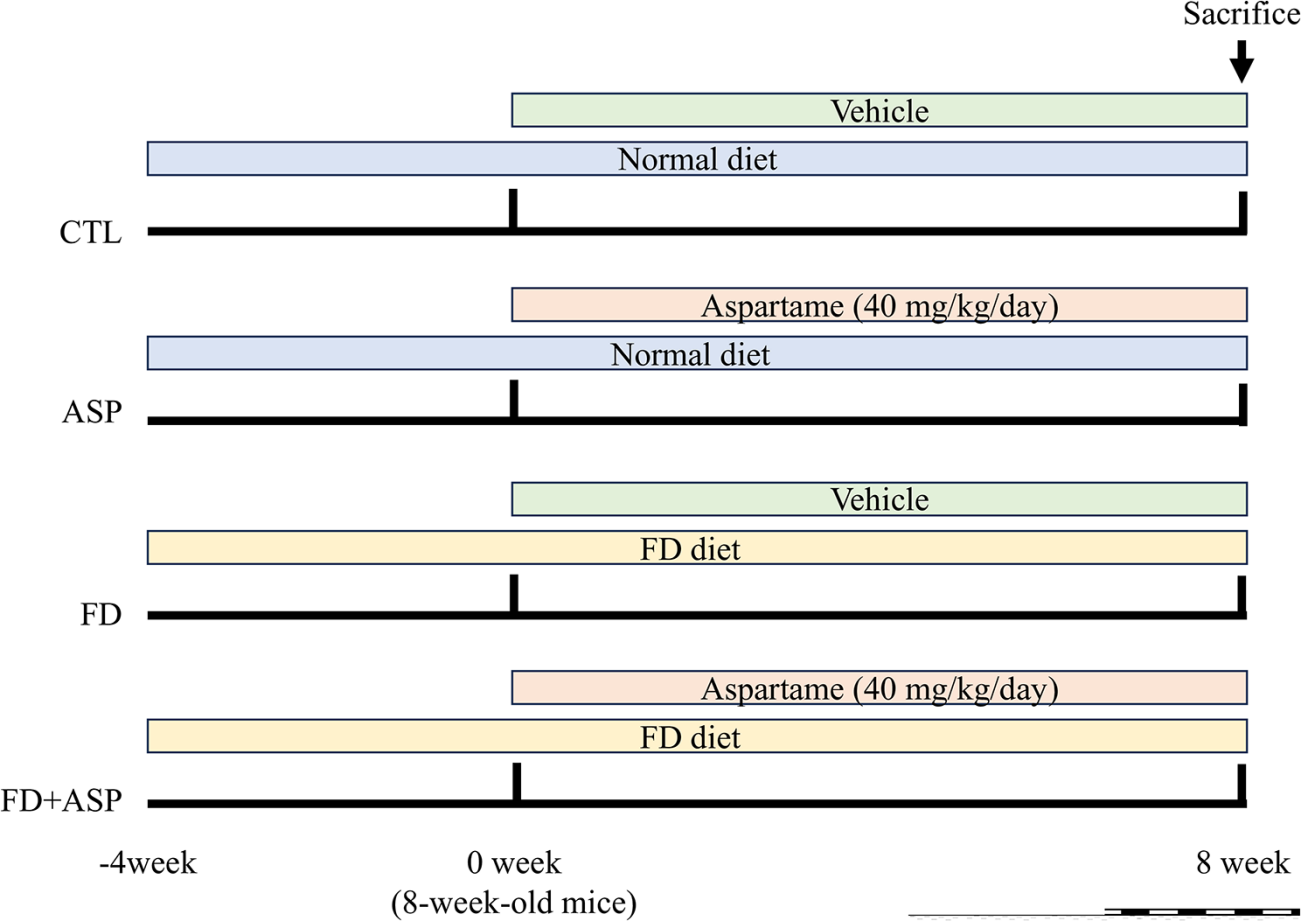
Experimental time course. CTL = control; ASP = aspartame administration; FD = folate-deficient diet; FD + ASP = aspartame-administration with a folate-deficient diet

The ICR mice were divided into four groups: (1) the control group (CTL), which included mice orally administered with 0.008 ml/g saline and fed a normal diet (*n* = 5); (2) aspartame group (ASP), which included mice orally administered with 40 mg/kg aspartame dissolved in saline and fed a normal diet (*n* = 5); (3) folate-deficient group (FD), which included mice orally administered with 0.008 ml/g saline and fed a folate-deficient diet (*n* = 5); and (4) folate-deficient with aspartame-treated group (FD + ASP), which included mice orally administered with 40 mg/kg aspartame dissolved in saline and fed a folate-deficient diet (*n* = 6). A folate-deficient diet was initiated four weeks before aspartame administration (Fig. 1). Eight weeks after treatment, 24-hour urine samples were collected, and body weights were measured. Subsequently, blood samples and kidneys were collected under anesthesia, and the mice were sacrificed. Animals were sacrificed by giving an overdose of isoflurane in accordance with regulations of animal experiments at Nagasaki University. Blood samples were collected in blood collection tubes without heparin. Serum was obtained through centrifugation (3000 rpm) of blood samples at 4 °C for 5 min (Cat. no. RL-120; Tomy Seiko, Tokyo, Japan). Serum creatinine (Cr), serum blood urea nitrogen (BUN), urinary *N*-acetyl-beta-glucosaminidase (NAG), urinary protein, and urinary Cr levels were measured using an enzymatic method by a commercial laboratory (SRL Inc, Tokyo, Japan). Dissected kidneys were fixed with 4% paraformaldehyde in phosphate-buffered saline (PBS; pH 7.4) immediately after sampling and embedded in paraffin. For histological analysis of the kidney, 3-µm-thick paraffin-embedded tissues were stained with a periodic acid-Schiff stain.

### Immunohistochemistry

Paraffin-embedded tissue sections were immunohistochemically examined using an indirect method. The following antibodies were used for immunohistochemistry: mouse anti-4-hydroxy-2-nonenal (4-HNE; 1:50; MHN-100P; JaICA, Shizuoka, Japan), which was used as an oxidative stress marker, and rabbit anti-superoxide dismutase 2 (SOD2; 1:100; ab13534; Abcam, Cambridge, UK), which was used as an antioxidant marker.

After deparaffinization, the sections were treated in an autoclave for 10 min at 120 °C for antigen retrieval. The sections were then treated with 0.3% H_2_O_2_ in methanol for 20 min to inactivate endogenous peroxidase activity. Thereafter, the sections were incubated for 30 min with a blocking solution at room temperature (RT). The sections were then incubated with the primary antibody diluted in the blocking solution overnight at 4 °C. Sections were then incubated with horseradish peroxidase-conjugated goat anti-rabbit immunoglobulin (P0448; Dako, Carpinteria, CA) or goat anti-mouse immunoglobulin antibodies (P0447; Dako, Carpinteria, CA) diluted at 1:100 or 1:200 for 1 h at RT. Reaction sites were visualized by treating the sections with H_2_O_2_ and 3,3′-diaminobenzidine tetrahydrochloride. After counterstaining with hematoxylin, the sections were dehydrated and mounted. For all specimens, negative controls were prepared using normal IgG instead of the primary antibody.

### Histological analysis

The image was transformed into a matrix of 1440 × 1024 pixels and viewed at 200 × or 400 × magnification using a light microscope (Nikon ECLIPSE Ci-L; Nikon, Tokyo, Japan). For semiquantitative evaluation of the positive areas for SOD and 4-HNE staining, DAB-positive areas were analyzed using the ImageJ FIJI software [11]. Five areas were selected for each sample, and the positive areas were determined at a × 200 magnification.

### Statistical analyses

Data are expressed as the mean ± standard error. Differences between the groups (CTL vs. ASP or FD vs. FD + ASP) were examined for statistical significance using the Student’s t-test. All statistical analyses were performed using JMP version 16 software (SAS Institute Inc., Cary, NC, USA). Statistical significance was set at *p* < 0.05.

## Results

### Aspartame did not induce renal histological changes and renal dysfunction in mice

**Fig. 2:**
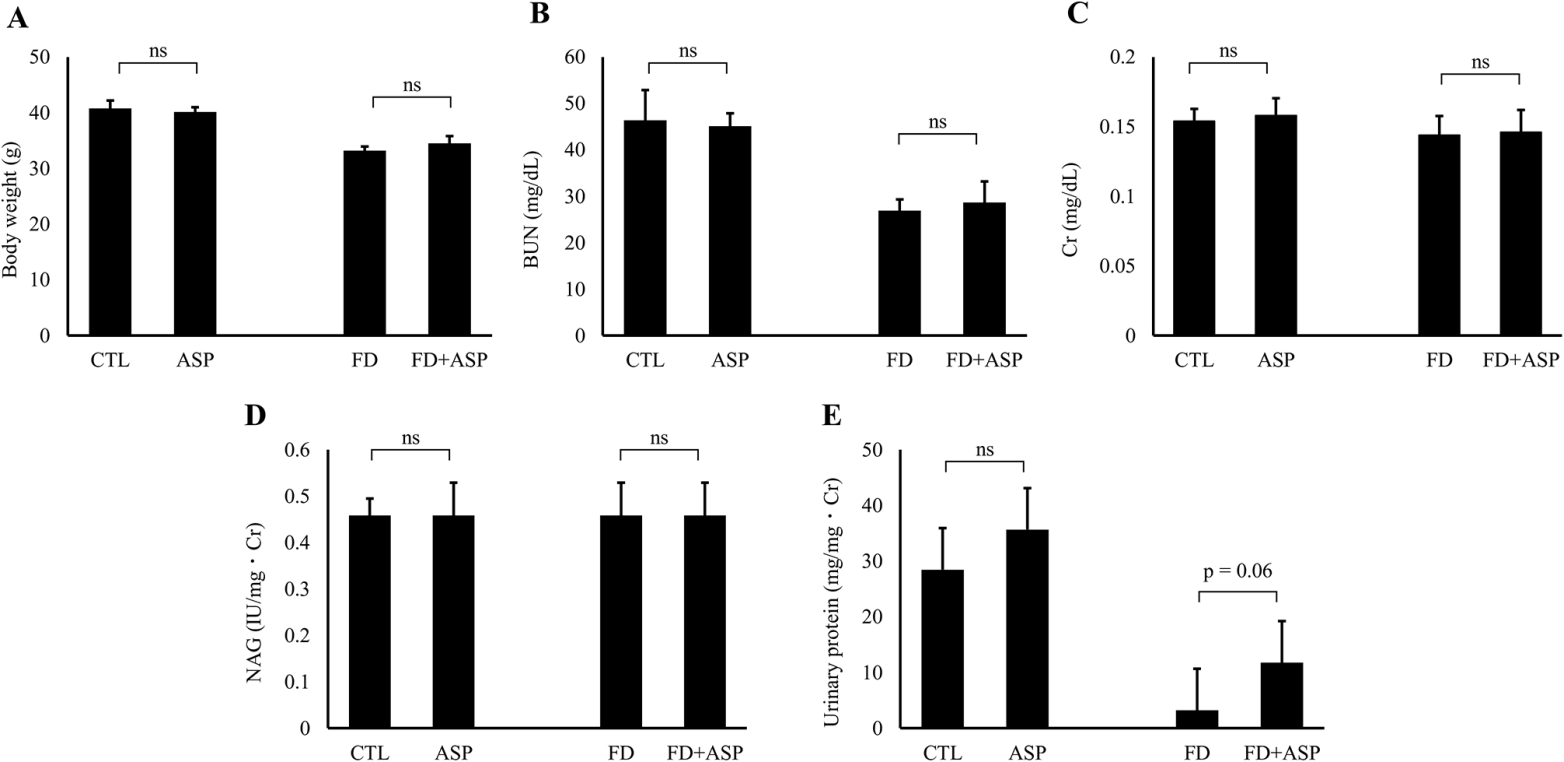
Evaluation of body weight, renal function, and urinary biomarkers. (**A**) Body weights of mice after an eight-week-long administration of aspartame. Body weights measured were similar between the CTL and ASP groups and between the FD and FD + ASP groups. (**B** and **C**) Serum blood urea nitrogen (BUN) and creatinine (Cr) levels of mice after eight weeks of aspartame administration. Both serum BUN and Cr levels were similar between the CTL and ASP groups and between the FD and FD + ASP groups. (**D** and **E**) Urinary *N*-acetyl-beta-glucosaminidase (NAG) and protein levels of mice after eight weeks of aspartame administration. Urinary NAG levels were similar between the CTL and ASP groups and between the FD and FD + ASP groups. Urinary protein levels were similar between the CTL and ASP groups, but higher levels were observed in the FD + ASP group than in the FD group. (**A**-**E**) *n* = 5 ∼ 6, each group. *p < 0.05; Student’s t-test; error bars indicate the mean ± standard error. Mouse test groups: CTL = control; ASP = aspartame-administered; FD = folate-deficient diet; FD + ASP = aspartame-administered with folate-deficient diet

**Fig. 3:**
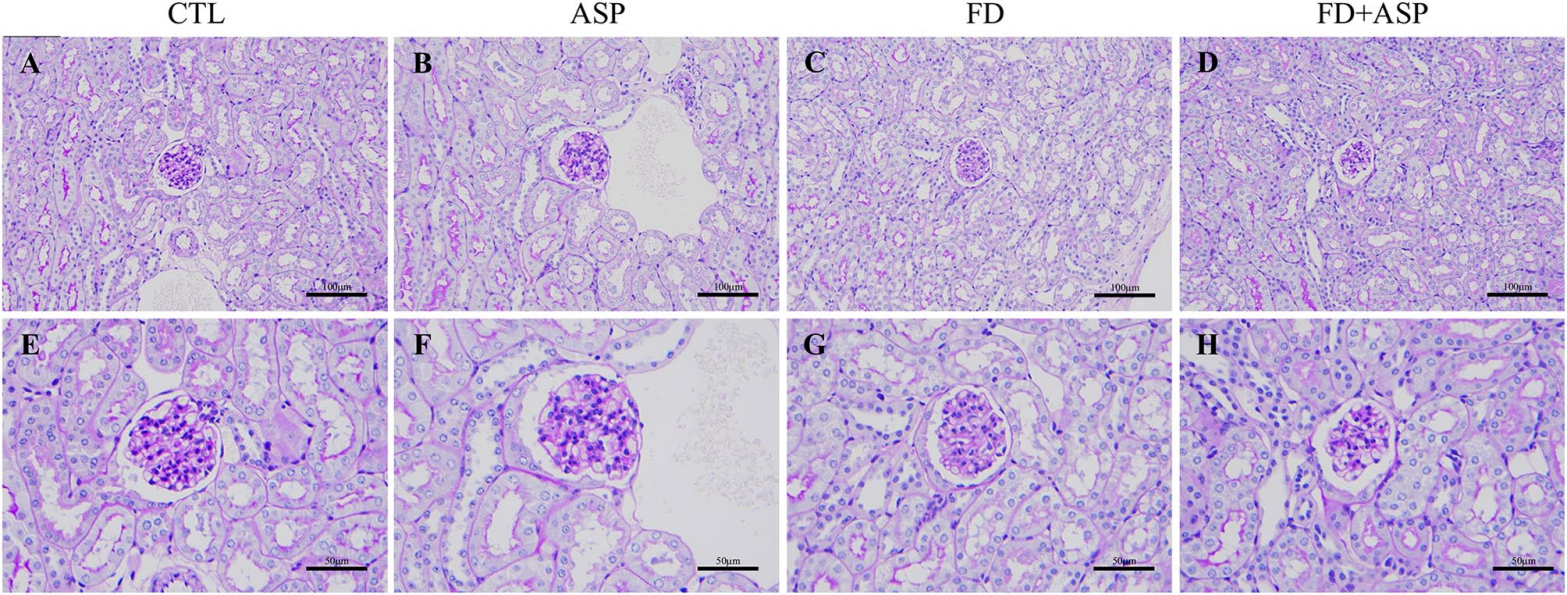
Evaluation of morphological changes in the kidneys. (**A**-**H**) No histological changes were observed in the glomerulus and tubulointerstitial lesions in any group. (**A**-**H**) Periodic acid-Schiff stain of the kidney at (**A**-**D**) ×200 and (**E**-**H**) ×400 magnifications. Mouse test groups: CTL = control; ASP = aspartame-administered; FD = folate-deficient diet; FD + ASP = aspartame-administered with folate-deficient diet

After eight weeks of aspartame administration, mice body weights were similar between the CTL and ASP groups and between the FD and FD + ASP groups (Fig. 2A). Serum BUN and Cr levels were also similar between the CTL and ASP groups and between the FD and FD + ASP groups (Fig. 2B and C). Furthermore, we analyzed urinary NAG as a marker of renal tubular injury and urinary protein as a marker of glomerular damage. Urinary NAG levels were similar between the CTL and ASP groups and between the FD and FD + ASP groups (Fig. 2D), and urinary protein levels were similar between the CTL and ASP groups but tended to be higher in the FD + ASP group than in the FD group (*p* = 0.06). However, urinary protein levels in the FD and FD + ASP groups were much lower than those in the CTL group, which was a normal control (Fig. 2E). In the ASP and FD + ASP groups, which were administered aspartame, histological changes in the glomerulus and interstitium were not observed and were similar to those in the CTL group (Fig. 3). These results suggest that an eight-week-long administration of 40 mg/kg aspartame did not induce renal histological changes or renal dysfunction in ICR mice.

### Aspartame did not induce renal oxidative stress in mice

**Fig. 4:**
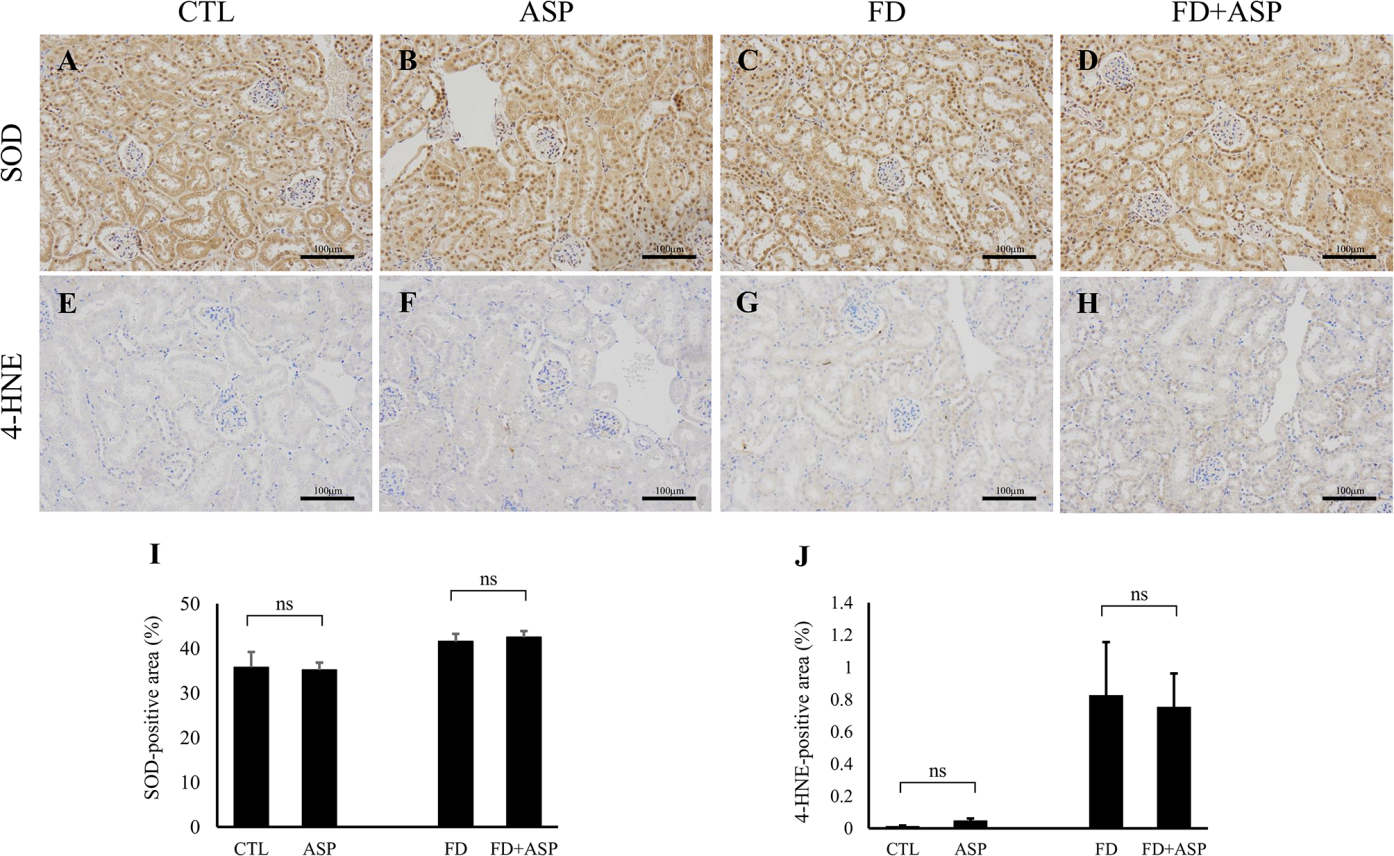
Immunohistochemistry for superoxide dismutase and 4-hydroxy-2-nonenal in the kidney. (**A**-**D**) Immunohistochemical analysis of superoxide dismutase (SOD) in the kidney (×200 magnification). SOD expression levels were similar between the CTL and ASP groups and between the FD and FD + ASP groups. (**I**) Bar graph showing the SOD-positive area. (**E**-**H**) Immunohistochemical analysis of 4-hydroxy-2-nonenal (4-HNE) in the kidney (×200 magnification). 4-HNE expression levels were similar between the CTL and ASP groups and between the FD and FD + ASP groups. (**J**) Bar graph showing the 4-HNE-positive area. (**I** and **J**) *n* = 5 ∼ 6, each group. *p < 0.05; Student’s t-test; error bars indicate the mean ± standard error. Mouse test groups: CTL = control; ASP = aspartame-administered; FD = folate-deficient diet; FD + ASP = aspartame-administered with folate-deficient diet

Although renal dysfunction and histological changes were not observed in the ASP and FD + ASP groups, previous studies have shown that aspartame reduces renal antioxidant capacity and increases renal oxidative stress. Therefore, we performed immunohistochemical analysis of SOD and 4-HNE as indicators of antioxidant capacity and oxidative stress, respectively. SOD was mainly expressed in renal tubular cells, and its expression levels were similar between the CTL and ASP groups and between the FD and FD + ASP groups (Fig. 4A-D and I). In contrast, the expression of 4-HNE was weakly expressed in renal tubular cells, and its expression levels were similar between the CTL and ASP groups and between the FD and FD + ASP groups (Fig. 4E-H and J). These results suggest that an eight-week-long administration of 40 mg/kg aspartame does not reduce renal antioxidant capacity or induce oxidative stress in ICR mice.

## Discussion

In the present study, no histological changes in mouse kidneys or renal dysfunction were observed when aspartame was administered at 40 mg/kg/day for eight weeks, including in mice that were fed a folic acid-deficient diet. Aspartame also did not induce oxidative stress in the kidneys. These results differ from those of previous animal studies.

Many animal studies have reported that aspartame affects kidneys [12–26]. These studies showed impaired renal function, decreased antioxidant levels, increased oxidative stress in renal tissue, and histological changes in the glomeruli, tubules, and interstitium. In particular, the reduction of antioxidants such as SOD is involved in renal oxidative stress [12, 18]. However, many studies have administered aspartame at doses far above the human ADI of 40–50 mg/kg/day [13, 15, 19–26]. Animal experiments using high doses of aspartame are useful in considering the possible mechanism of its toxicity; however, whether aspartame is associated with renal dysfunction in a clinical setting remains unclear.

In this study, we investigated the effects of using aspartame at 40 mg/kg/day on mouse kidneys, which is the ADI set by the European Union [4]. Previous research has shown that the intake of aspartame by the general population falls below 50 mg/kg/day, and we determined 40 mg/kg/day as a realistic value [27, 28]. This study differs from others in that the mice were fed a folic acid-deficient diet. This is because, humans have less folate in the liver than rodents; thus, humans are more likely to metabolize methanol via an alternate pathway (the microsomal pathway) [9]. Many previous studies did not use folate deficiency models and may not mimic clinical settings. In this study, we examined the nephrotoxicity of aspartame under conditions closer to those of clinical settings for dosage and folic acid deficiency. Overall, aspartame had no obvious effects on mouse kidneys, including antioxidative capacity (SOD) and oxidative stress (4-HNE).

However, a small number of animal studies have demonstrated aspartame-induced renal damage using an approach similar to that used in the current study. For example, Kumar et al. reported that the administration of 40 mg/kg/day aspartame for 30 days to rats fed a folate-deficient diet increased oxidative stress in the kidneys and serum Cr and BUN levels [17], which is inconsistent with our results. One of the differences in our study is the animal species used. Although many previous studies have used albino rats, we investigated the renal effects of aspartame in ICR mice. It cannot be ruled out that differences in the animals used in this study may have contributed to the differing results obtained in our study.

Furthermore, histological findings by Gabr et al. revealed glomerular and renal tubular damage in rats treated with 20 mg/kg/day aspartame for 180 days [14]. The current study tested aspartame administration for eight weeks; thus, the effects of long-term aspartame administration cannot be ruled out. Although this study alone cannot completely disprove that aspartame harms the kidneys in a near-clinical setting, our results indicate that aspartame might not affect kidneys and is consistent with a clinical trial [29].

In conclusion, our results show that aspartame does not affect the kidneys, in contrast to previous animal studies. Although our results are consistent with those of clinical studies, further basic and clinical research is needed to confirm whether aspartame is safe for the kidneys.

### Limitations

Although our study presents a different result from previous studies in that aspartame does not affect the kidneys and encourages consideration of its safety, it has several limitations. First, in this study, serum folate levels were not measured in the FD + ASP groups; therefore, whether aspartame was administered in a folate-deficient state is unclear. However, in our pilot study, we confirmed that feeding mice a folate deficiency diet for four weeks resulted in deficient serum folate levels (Supplemental Fig. 1), consistent with previous results [10]. The FD + ASP group was fed a folate-deficient diet for four weeks before aspartame administration, indicating that aspartame was likely administered in a folate-deficient state. Second, we used immunohistochemistry to evaluate oxidative stress, which may not have been easy to evaluate early or identify minor changes. Finally, we only confirmed the effects of aspartame on the kidneys, and it is unclear whether the aspartame doses tested are safe for other organs. Therefore, it cannot be concluded from this study alone that aspartame is safe for humans, and further research, including studies on organs other than the kidneys, is necessary.

### Electronic supplementary material

Below is the link to the electronic supplementary material.

Supplementary Material 1

## Data Availability

No datasets were generated or analysed during the current study.

## Abbreviations

4-HNE: 4-hydroxy-2-nonenal
ADI: Maximum daily intake
ASP: Aspartame-administered group
BSA: Bovine serum albumin
BUN: Blood urea nitrogen
Cr: Creatinine
CTL: Control group
FD: Folate-deficient diet group
FD + ASP: Aspartame-administered with a folate-deficient diet group
NAG: N-acetyl-beta-glucosaminidase
PBS: Phosphate-buffered saline
RT: Room temperature
SOD: Superoxide dismutase
