# Supplementary figures and images for: Aspartame, as an artificial sweetener, does not affect renal function and antioxidative states in mice
Source: BMC Res Notes. 2024 Jun 5;17:155. doi: 10.1186/s13104-024-06816-6 (PMC11155020; doi:10.1186/s13104-024-06816-6)

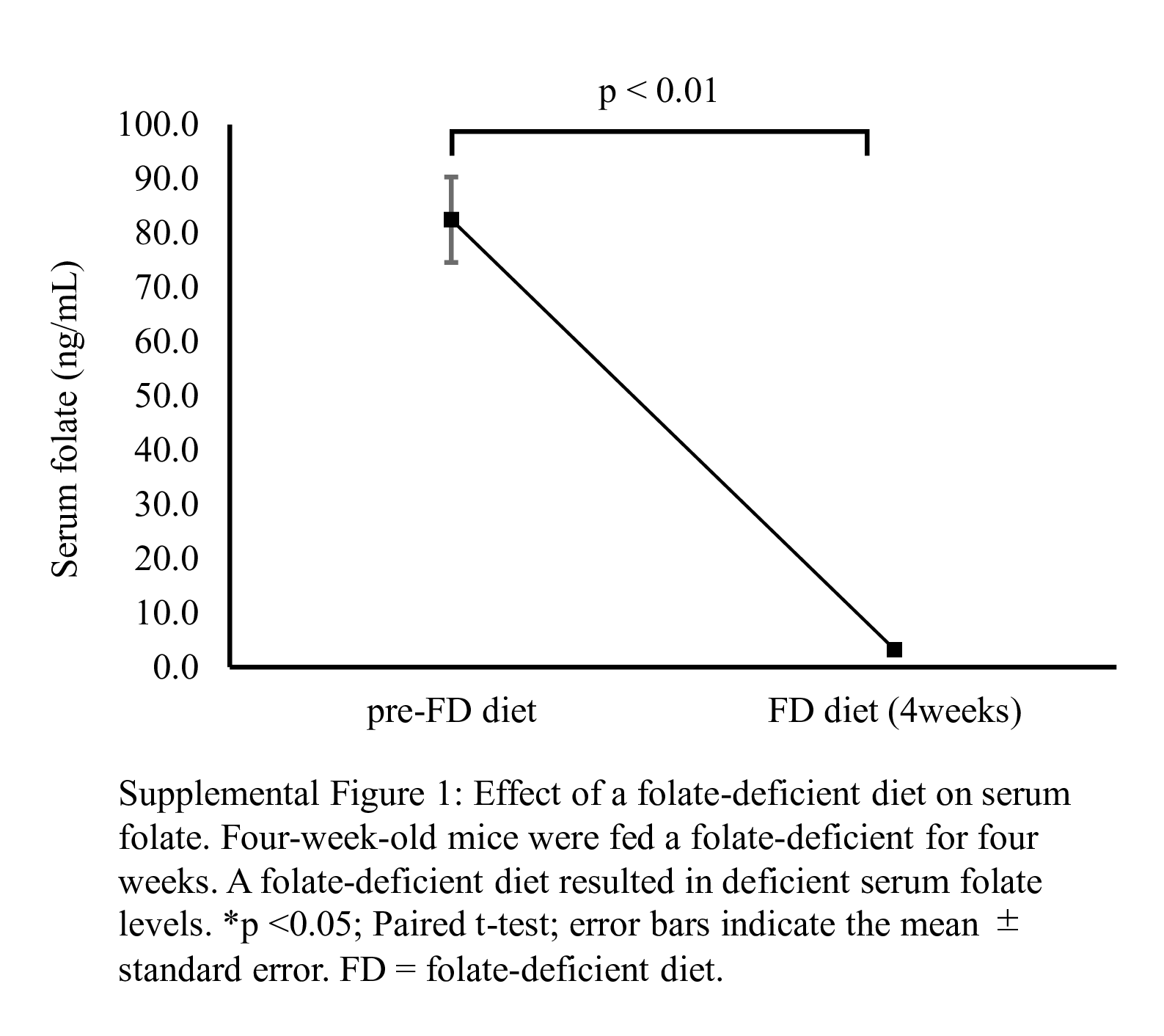

Supplement: Supplementary file 1 — Supplementary Material 1 [file 13104_2024_6816_MOESM1_ESM.tiff]
